# The Diagnostic Usefulness of Serum Total Bile Acid Concentrations in the Early Phase of Acute Pancreatitis of Varied Etiologies

**DOI:** 10.3390/ijms18010106

**Published:** 2017-01-06

**Authors:** Aleksandra Maleszka, Paulina Dumnicka, Aleksandra Matuszyk, Michał Pędziwiatr, Małgorzata Mazur-Laskowska, Mateusz Sporek, Piotr Ceranowicz, Rafał Olszanecki, Marek Kuźniewski, Beata Kuśnierz-Cabala

**Affiliations:** 1 Department of Diagnostics, University Hospital, 31-501 Kraków, Poland; amaleszka@su.krakow.pl (A.M.); mbmazur@cyf-kr.edu.pl (M.M.-L.); 2 Department of Medical Diagnostics, Jagiellonian University Medical College, 30-688 Kraków, Poland; paulina.dumnicka@uj.edu.pl; 3 Department of Anatomy, Jagiellonian University Medical College, 31-034 Kraków, Poland; aleksandra.matuszyk@uj.edu.pl (A.M.); msporek1983@gmail.com (M.S.); 4 2nd Department of Surgery, Jagiellonian University Medical College, 31-501 Kraków, Poland; michal.pedziwiatr@uj.edu.pl; 5 Surgery Department, The District Hospital, 34-200 Sucha Beskidzka, Poland; 6 Department of Physiology, Jagiellonian University Medical College, 31-531 Kraków, Poland; 7 Department of Pharmacology, Jagiellonian University Medical College, 31-531 Kraków, Poland; rafal.olszanecki@uj.edu.pl; 8 Chair and Department of Nephrology, Jagiellonian University Medical College, 31-501 Kraków, Poland; marek.kuzniewski@uj.edu.pl; 9 Department of Diagnostics, Chair of Clinical Biochemistry, Jagiellonian University Medical College, 31-501 Kraków, Poland; mbkusnie@cyf-kr.edu.pl

**Keywords:** idiopathic acute pancreatitis, total bile acids, biliary acute pancreatitis

## Abstract

The most common causes of acute pancreatitis (AP) are biliary tract diseases with cholestasis and alcohol consumption. In 10%–15% of patients, etiology determination is difficult. Identification of the etiology allows for the implementation of adequate treatment. The aim of this study was to assess the utility of the serum concentrations of total bile acids (TBA) to diagnose AP etiology in the early phase of the disease. We included 66 patients with AP, admitted within the first 24 h from the onset of symptoms. TBA were measured in serum at 24, 48, and 72 h from the onset of AP, using an automated fifth generation assay. The bilirubin-to-TBA ratio (B/TBA) was calculated. TBA was highest on the first day of AP and decreased subsequently. In patients with biliary etiology, serum TBA was significantly higher compared to those with alcoholic and other etiologies. B/TBA was significantly higher in patients with alcoholic etiology. At admission, the cut-off values of 4.7 µmol/L for TBA and 4.22 for the B/TBA ratio allowed for a differentiation between biliary and other etiologies of AP with a diagnostic accuracy of 85 and 83%. Both TBA and B/TBA may help in the diagnosis of AP etiology in the early phase of AP.

## 1. Introduction

Acute pancreatitis (AP) is a severe disease with high mortality and uncertain prognosis. The reason for this is its diverse etiology and the unpredictable course of the disease. Oxidative stress, inflammation, and other pathophysiological processes, such as apoptosis and necrosis, have been related with AP and are accountable for morphological changes of the pancreas in the progress of AP [1]. The etiology, pathogenesis, and progression of acute pancreatitis are the subjects of numerous experimental and clinical studies [2]. Autodigestion of pancreatic tissue, which is the reason for AP, is a process dependent on early activation of inert zymogens into active digestive enzymes. In AP, digestive enzymes work not only within the pancreatic tissue, but also penetrate into blood vessels and are transported through the bloodstream to all body tissues [3]. The literature describes over 80 factors responsible for the development of AP [4,5]. The etiology of AP, apart from alcohol abuse and cholelithiasis, may also include a vascular component responsible for pancreatic ischemia [6]. However, the most frequent etiology is cholelithiasis, which accounts for 30%–60% of cases [4,7,8,9,10]. The majority of cases of cholelithiasis are discovered during ultrasound examination of the abdomen, but detecting minor deposits in the biliary duct or peripapillary changes (e.g., tumors) is possible thanks to endoscopic ultrasound examination (EUS). Since imaging techniques have improved, enabling the detection of micro-cholelithiasis and the so-called biliary sludge (a term used in exchange with micro-cholelithiasis), the percentage of patients with undiagnosed etiology, or idiopathic AP, has decreased considerably [9,10,11].

Recent studies indicate that micro-cholelithiasis may induce AP regardless of the presence of macroscopically-visible gallstones [11]. It has been demonstrated that micro-cholelithiasis, the sphincter of Oddi dysfunction (SOD), anatomical dysfunctions in pancreatic morphology, and genetic mutations are among the main causes of AP initially recognized as idiopathic [8,10,12,13,14]. Among the examinations recommended to exclude micro-cholelithiasis are microscopic examinations of bile and EUS. Currently, with regard to non-invasive imaging, magnetic resonance cholangiopancreatography (MRCP) plays a considerable role [7,10,15].

Cholelithiasis develops when the normal ratio of bile acids to cholesterol concentration in bile is disturbed [11,16,17,18]. Acids that arise from cholesterol as a precursor are called primary bile acids; among them, the most important ones are cholic acid and chenodeoxycholic acid. Under the influence of intestinal bacteria, primary bile acids undergo dehydroxylation and deconjugation, transforming into secondary and tertiary bile acids. The main secondary bile acids in humans are deoxycholic and lithocholic acids, and the main tertiary bile acids are ursodeoxycholic and sulpholithocholic [16]. The reserve of bile acids in physiological conditions is relatively constant and is subject to continuous intestinal-hepatic circulation. Blood concentrations of bile acids are considered indicators of hepatic activity. Within 24 h, the liver removes about 20 g of bile salts from the blood.

Until recently, the concentrations of bile acids could only be measured in the bile by the expensive and specialist method of high-performance liquid chromatography (HPLC). For a few years, a routinely applied diagnostic test has been available, which allows for a measurement of the total amount of bile acids in serum (TBA). A TBA test of the fifth generation is included in the so-called “liver panel” and is considered to be a sensitive test that allows for early diagnosis of hepatic dysfunctions, before symptoms of the disease occur (e.g., icterus). The TBA test is commonly considered to be prognostic in patients treated for hepatitis C infection, in pregnant women with suspected cholestasis, and in veterinary medicine. Moreover, an increase in fasting serum TBA may be related to alcoholic liver disease, hepatic damage induced by chemical substances and medical drugs, cystic fibrosis, cirrhosis, or primary hepatic carcinoma.

Serum concentrations of bile acids, which are the main ingredient of bile, increase considerably in patients with cholelithiasis. In physiological conditions, bile acids do not have access to pancreatic cells. This only occurs in the course of cholestasis of the bile ducts. The studies on animal models have found that bile acids may damage pancreatic cells and initiate inflammatory processes in this organ [19,20].

The aim of the present study was to assess the serum concentrations of TBA with the fifth generation test in patients with AP of varied etiology and to evaluate the diagnostic usefulness of such measurements for the early diagnosis of AP etiology, in particular, the biliary etiology of AP.

## 2. Results

### 2.1. Differences Related to Acute Pancreatitis Etiology

The majority of studied patients were diagnosed with AP of biliary etiology (Table 1). There were no significant differences regarding AP severity between the groups with varied etiology, although the percentage of patients with mild acute pancreatitis (MAP) was the highest among those whose AP was of biliary origin and the percentage of patients with severe acute pancreatitis (SAP) was the highest among patients with alcoholic etiology (Table 1). Patients with alcoholic etiology of AP were hospitalized the longest (i.e., 14 ± 12 days). These patients were also significantly younger than the patients with biliary etiology. The prevalence of comorbidities was higher among patients with biliary AP. Additionally, serum bilirubin concentrations, and the activities of alanine and aspartate aminotransferases (ALT and AST, respectively), alkaline phosphatase (ALP), and γ-glutamyl transferase (GGT), were higher in this group (Table 1).

### 2.2. Changes in Total Bile Acid Concentrations and Serum Bilirubin-to-Total Bile Acids Ratio during the First 72 h of Acute Pancreatitis of Various Etiologies

Increased serum concentrations of TBA were observed during the first 24 h from the onset of symptoms in all patients with AP and equaled 64.1 ± 79.8 µmol/L. On subsequent days, a statistically significant decrease in TBA concentrations was observed: on day 2 of AP, mean serum TBA was 24.0 ± 61.6 µmol/L; on day 3, it decreased to 11.5 ± 27.8 µmol/L (*p* < 0.001).

**Figure 1:**
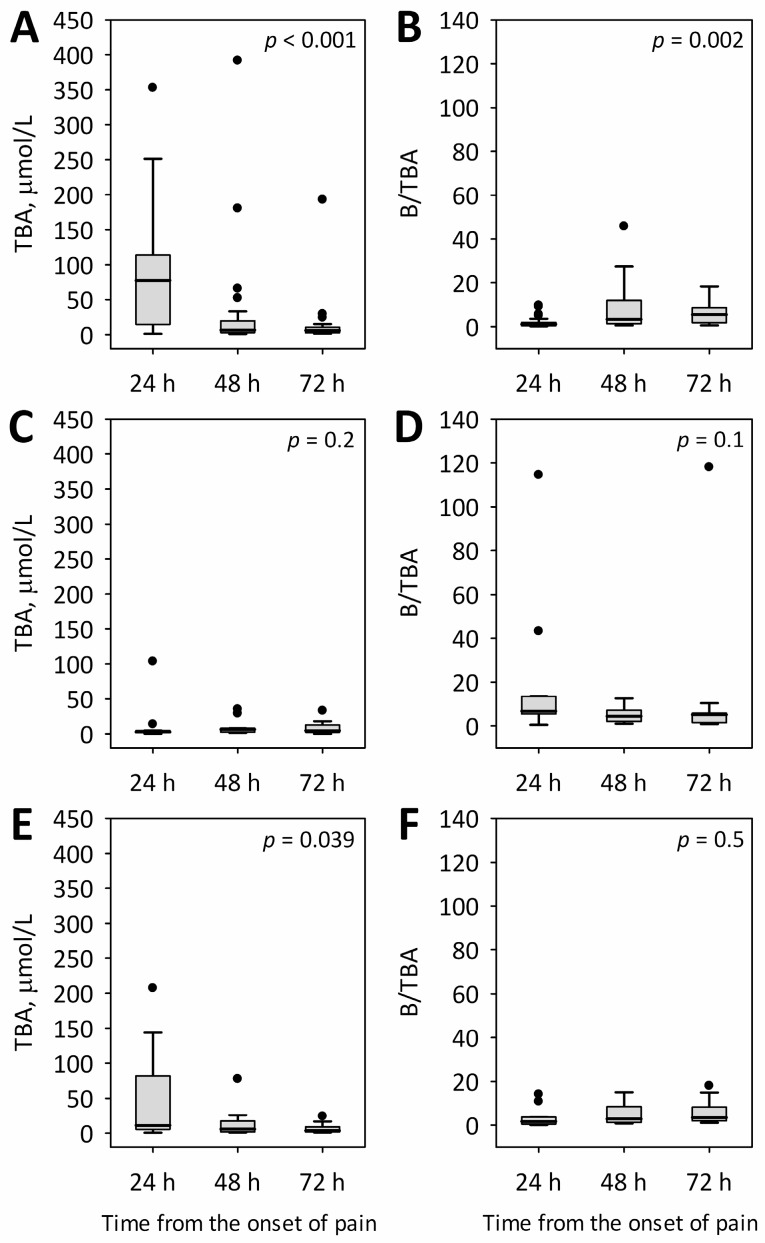
Changes in TBA serum concentrations and B/TBA ratios in patients with acute pancreatitis of biliary (**A**,**B**); alcoholic (**C**,**D**); and other (**E**,**F**) etiologies during the first 72 h of the disease. Data are shown as median, interquartile range (box), non-outlier range (whiskers), and outliers (dots); *p*-values for the difference between the time points are shown on the graphs.

On day 1 of AP, average serum concentrations of TBA were the highest in the group with biliary etiology of AP and were nearly six times higher compared to the group with alcoholic etiology and twice as high compared to the patients with AP due to other etiologic factors (Table 1). On subsequent days, TBA concentrations in the group of patients with biliary etiology decreased to 32.4 ± 78.7 µmol/L on day 2 and 14.2 ± 35.6 µmol/L on day 3 of AP (*p* < 0.001; Figure 1A). In patients with alcoholic etiology, the mean TBA concentrations normalized and equaled 10.5 ± 12.6 µmol/L on day 2 and 9.1 ± 10.6 µmol/L on day 3; however, no statistically significant changes were observed during the first 72 h of AP (Figure 1C). In patients with etiologies other than biliary or alcoholic, a decrease and normalization of TBA concentrations were observed during the study (Figure 1E).

Additionally, we calculated the ratio of serum bilirubin-to-TBA concentrations (B/TBA ratio). This ratio was significantly higher in patients with alcoholic etiology of AP in comparison to those with biliary etiology and to the remaining patients with other etiologies of AP (Table 1). The mean values of B/TBA in patients with biliary etiology was four times lower and, in the group with other etiologies, six times lower as compared to the patients with alcoholic AP (Table 1). In AP of biliary origin, the values of B/TBA were the lowest on day 1 of AP, and were significantly higher on subsequent days of the study (*p* = 0.002; Figure 1B). In contrast, no significant time changes in B/TBA were observed among patients with alcoholic (Figure 1D) and other (Figure 1F) etiologies.

### 2.3. The Relationships between Total Bile Acid Concentrations and the Markers Related to Etiology and Severity of Acute Pancreatitis

On the first day of the study, positive correlations were observed between serum TBA and bilirubin as well as the activities of ALT, AST, ALP, GGT, and amylase in the whole studied cohort. In the case of the B/TBA ratio, these relationships were negative (Table 2).

**Figure 2:**
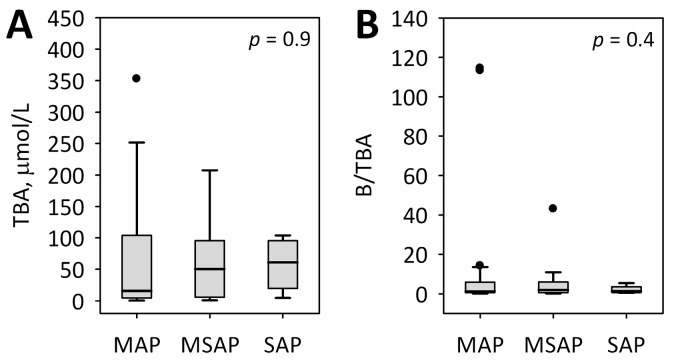
TBA serum concentrations (**A**); and B/TBA ratio (**B**) at 24 h from the onset of AP symptoms among patients with MAP, MSAP, and SAP, irrespective of etiology. Data are shown as median, interquartile range (box), non-outlier range (whiskers), and outliers (dots).

There were no significant relationships between TBA and the severity of AP (Figure 2). In logistic regression adjusted for etiology, TBA or B/TBA at any time point were not associated with severe or moderately-severe AP (at 24 h from the onset of AP: odds ratio 1.00; 95% confidence interval 0.99–1.01 per 1 µmol/L increase in TBA; *p* = 0.8; odds ratio 0.98; 95% confidence interval 0.94–1.03 per one unit increase in B/TBA; *p* = 0.4). No correlations were observed between TBA or B/TBA and the bedside index for severity in AP (BISAP) score [21]. Additionally, TBA concentrations did not correlate with inflammatory markers—i.e., C-reactive protein (CRP) and leukocyte count (WBC).

### 2.4. The Diagnostic Accuracy of Total Bile Acid Concentrations and Bilirubin-to-Total Bile Acids Ratio for Differentiating between Biliary and Other Etiologies of Acute Pancreatitis

**Figure 3:**
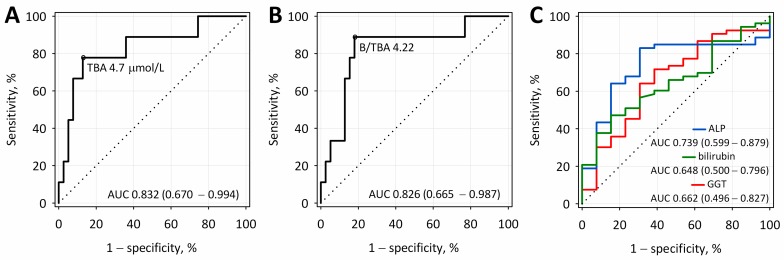
Receiver operating characteristic (ROC) curves for TBA (**A**); and B/TBA (**B**) for the diagnosis of biliary etiology in patients with acute pancreatitis at 24 h from the onset of symptoms. For comparison, ROC curves are presented for ALP, bilirubin, and GGT measured at the same time point (**C**). AUC: area under the ROC curve.

The accuracy of serum TBA and B/TBA ratio to diagnose AP of biliary etiology was assessed with receiver operating characteristic (ROC) curve analysis. Diagnostic sensitivity, specificity, accuracy, as well as the positive (PPVs) and negative predictive values (NPVs), were calculated for the selected cut-off points (Table 3). The values of area under the ROC curve (AUC) were above 0.8 for both TBA and the B/TBA ratio measured on the first day of AP and did not differ significantly (Figure 3). The cut-off values of 4.7 µmol/L for TBA and 4.22 for the B/TBA ratio enabled a differentiation between biliary and non-biliary etiologies of AP with the diagnostic accuracy comparable to ALP and considerably higher than serum total bilirubin and GGT (Figure 3, Table 3).

## 3. Discussion

In the present study, serum TBA concentrations were measured using the routinely available, automated fifth generation test. We observed increased TBA concentrations in the whole studied group of patients with AP. The dynamics of changes in TBA concentrations during the first 72 h of AP were assessed: the highest concentrations of TBA were observed on the first day of AP, followed by a significant decrease on subsequent days. The highest concentrations of serum TBA were associated with biliary etiology of AP. In patients with this etiology, mean serum TBA was above upper reference limit during the whole study. Additionally, statistically significant correlations were shown between increased serum TBA and the results of other biochemical tests—i.e., total bilirubin, enzymes related to cholestase (ALP, GGT), and hepatic transaminases (ALT, AST). In addition to TBA concentrations, a ratio of bilirubin to TBA may be of practical value, as it was significantly increased in patients with alcoholic etiology of AP. 

Pancreatitis is caused by inflammatory injury to the exocrine pancreas, and the recovery is achieved via regeneration of digestive enzyme-producing acinar cells [22]. Although the clinical picture of AP is independent of the triggering etiological factor, there are several etiology-specific issues regarding diagnostic and therapeutic procedures, especially in those patients who develop more severe AP [23]. Most importantly, there is a possibility of causative treatment of biliary AP. According to the recommendations of the American Gastroenterological Association (AGA) issued in 2013, in patients with co-existing cholestasis of biliary origin, it is recommended that endoscopic retrograde cholangiopancreatography (ECPW) be performed during the first 24 h of AP [24]. 

With regard to laboratory tests in the early phase of AP, in addition to pancreatic enzymes (lipase, amylase), it is recommended that the hepatic function with the use of hepatic enzymes (such as ALT, AST, ALP, GGT), total serum bilirubin, and hepatic tumor markers (α-fetoprotein) be assessed [23]. It was observed that at least a three-fold increase in transaminases’ activities above the upper normal limit allows for the prediction of a biliary etiology of AP with a PPV of 95% [23]. The relationship between increased activity of aminotransferases and the biliary etiology of AP was first described in 1979 by McMohon and Picford; since then, aminotransferases have been considered the most useful hepatic markers measured in patients with biliary AP [25]. However, in the present study, the suggested three-fold increase in ALT and AST activity was observed on the first day of AP regardless of etiology (Table 1). 

The study of Liu et al. indicates that EUS together with aminotransferases has a considerably higher prognostic value during the first 24 h from the admission of a patient with AP [26]. Similar conclusions were presented in the study by Gungor et al. [15]. On the other hand, Levy et al. demonstrated that the independent factors associated with biliary etiology were female sex, age above 58 years and ALT activity at admission above 150 U/L [27]. This is consistent with our data where approximately 60% of patients with biliary etiology were female and the average age in this group was significantly higher in comparison with alcoholic AP.

In our study, the diagnostic accuracy of TBA and B/TBA for the detection of biliary etiology was comparable to the diagnostic accuracy of ALP and higher than the accuracy of bilirubin and GGT. In each etiologic group, an increase in bilirubin, ALP, and GGT activity was demonstrated; however, in spite of significantly higher values in the group with biliary AP, bilirubin concentrations, ALP, and GGT activities did not show statistically significant differences between patients with alcoholic and other etiologies of AP. In the case of hepatic damage, an increase in serum TBA may precede an increase in GGT activity.

Considering the possibilities of causative treatment, the early detection of biliary AP seems especially important. In our study, TBA concentrations and B/TBA ratios allowed for discrimination between biliary and non-biliary (in particular alcoholic) AP at 24 h from the onset of AP symptoms. In this aspect, our results are promising. It may be helpful to include serum TBA in the panel of laboratory tests performed in AP patients at admission. The TBA test is routinely available and relatively inexpensive, and its results can be obtained in just a few hours after a doctor’s order. However, our results must be considered preliminary, because of a limited number of patients recruited in a single center. Additionally, we could not identify any earlier studies that assessed TBA concentrations in patients in the early phase of AP with the use of a routine automated method. Therefore, we believe it is necessary to conduct further prospective studies on this subject with a larger patient sample.

## 4. Material and Methods

### 4.1. Patients and Laboratory Tests

The study included 66 patients hospitalized and treated because of AP in the Surgical Ward of the District Hospital in Sucha Beskidzka, Poland, admitted within first 24 h from the onset of symptoms of AP. The diagnosis of AP was done in compliance with the 2012 revision of Atlanta Classification [23,28]. The study group included 32 women and 34 men with a mean age of 61 ± 18 years. The mean time of hospitalization was 9 ± 8 days. Forty-six patients (70%) were diagnosed with MAP, 15 (23%) with MSAP, and 5 (8%) with SAP. Etiology was diagnosed based on clinical interview, physical examination, and imaging and laboratory tests on admission. Biliary etiology was diagnosed when gallstones were detected in transabdominal ultrasonography or computed tomography. Alcohol etiology was diagnosed when a patient had a history of significant alcohol consumption lasting for at least 5 years. Biliary etiology was found in 38 patients (58%) and alcoholic etiology was found in 13 patients (20%), and the remaining 15 patients (23%) were diagnosed with AP of another etiology, including hypertriglyceridemia in five patients, post-endoscopic retrograde cholangiopancreatography in one patient, and an unknown cause or idiopathic AP in nine patients. The characteristics of the studied group with respect to AP etiology are presented in Table 1. The diagnosis of preexisting cholelithiasis was based on interview.

The blood was collected from patients on the day of admission (about 24 h from the onset of symptoms of AP) and on two subsequent days (48 and 72 h from the onset of AP). The routine biochemistry panel (including serum concentrations of bilirubin, C-reactive protein, albumin, calcium, glucose, urea, creatinine, and serum activities of amylase, GGT, ALP, ALT, and AST) and the complete blood count were performed at Laboratory Diagnostic Department of the District Hospital in Sucha Beskidzka, Poland. Blood counts were performed in EDTA-anticoagulated whole blood. Biochemical tests were conducted in the sera obtained during centrifuging of blood collected without anticoagulant for 10 min at 4000 rpm on an MPW 351e centrifuge (MPW Med. Instruments, Warszawa, Poland; rotor No. 12436). Fifth generation Total Bile Acids Randox RX Series assay (Randox Laboratories, Crumlin, UK) was used to measure total concentrations of bile acids in sera. The measurements were conducted on a Cobas 8000 analyzer (Roche Diagnostics, Basel, Switzerland) in the Diagnostic Department of the University Hospital in Kraków, Poland. The assay was standardized against the Diazyme Total Bile Acids Assay (Diazyme, Poway, CA, USA) and Total Bile Acids Assay BQ Kit (Enzymatic Cycling, San Diego, CA, USA). The fifth generation test is the stable liquid test ready to use with all types of automatic clinical chemistry analyzers. A small sample size is necessary for testing (about 5 µL), and random samples may be used due to small influence of hemolysis and lipemia. The reference interval for TBA provided by the manufacturer of the assay was 2–10 µmol/L.

The participation of patients in the study was voluntary, and each patient signed the informed consent for the study. The study protocol was approved by the Bioethics Committee of the Jagiellonian University (approval No. KBET/247/B/2013; permission date 28 November 2013).

### 4.2. Statistical Analysis

The data was presented as the number of patients (the percentage of the group) for nominal variables and the mean ± standard deviation for continuous variables. The normality of distributions was assessed with a Shapiro–Wilk test. Differences between groups were analyzed with a chi-square test in the case of nominal data and with a one-way ANOVA or a Kruskal–Wallis ANOVA in the case of quantitative data with normal or non-normal distribution. Between-day changes in TBA concentrations were assessed with a Friedman test. Correlations were evaluated with the Spearman’s rank correlation coefficient. The assessment of diagnostic accuracy was based on ROC curves. The tests were two-tailed and the results were considered significant at *p* ≤ 0.05. The calculations were conducted with the Statistica 12.5 package (StatSoft Inc., Tulsa, OK, USA).

## 5. Conclusions

The introduction of improved imaging diagnostic modalities in the early phase of AP has changed the recommended panel of laboratory tests. However, the methods of imaging diagnostics are associated with a possibility of complications, often require sedation and an application of contrast agents, and are highly specialist examinations, expensive, and not always available. For this reason, there is still a need for non-invasive methods that would allow for an early diagnosis of etiology of AP. Our preliminary results indicate that the measurement of TBA in serum may be useful in the early phase of AP, especially in patients with unclear etiology. However, as our study included relatively small numbers of patients, the results should be confirmed in studies of a larger scale.

## Figures and Tables

**Table 1:** Clinical characteristics of patients and the results of laboratory tests at 24 h from the onset of acute pancreatitis with respect to the etiology.

	Biliary AP (*N* = 38)	Alcoholic AP (*N* = 13)	Other Etiology of AP (*N* = 15)	*p*	Reference Range ^1^
Age, years	65 ± 17	45 ± 19	66 ± 14.5	0.007	-
Duration of hospital stay, days	7 ± 3	14 ± 12	9 ± 8	0.6	-
Male sex, *N* (%)	15 (39)	13 (100)	6 (40)	0.005	-
MAP, *N* (%) MSAP, *N* (%) SAP, *N* (%)	28 (74) 8 (21) 2 (5)	8 (62) 3 (23) 2 (15)	10 (67) 4 (27) 1 (7)	0.8	-
Pre-existing comorbidities, *N* (%)	36 (95)	6 (46)	9 (60)	<0.001	-
Pre-existing cholelithiasis, *N* (%)	34 (89)	2 (13)	0	<0.001	-
Pancreatic necrosis, *N* (%)	0	2 (15)	1 (7)	0.064	-
Peripancreatic fluid collections, *N* (%)	0	2 (15)	3 (20)	0.022	-
Transient organ failure, *N* (%)	6 (16)	0	1 (7)	0.2	-
Persistent organ failure, *N* (%)	2 (5)	2 (15)	1 (7)	0.5	-
ERCP, *N* (%)	4 (11)	1 (8)	1 (7)	0.9	-
BISAP ≥ 3	3 (8)	2 (15)	1 (7)	0.7	-
Death, *N* (%)	1 (3)	1 (8)	1 (7)	0.7	-
TBA, µmol/L	86.6 ± 86.8	14.7 ± 33.5	47.0 ± 69.1	0.003	2–10
Bilirubin, µmol/L	59.2 ± 39.2	31.7 ± 19.5	33.6 ± 32.1	0.004	0–21
B/TBA ratio	5.95 ± 21.18	22.48 ± 36.75	3.49 ± 4.86	0.008	-
CRP, mg/L	37.0 ± 57.7	95.0 ± 108.1	56.674.5	0.1	0–5
Albumin, g/L	40.0 ± 5.3	39.4 ± 5.1	39.1 ± 5.0	0.8	35-52
WBC, ×10^3^/µL	13.3 ± 7.95	13.7 ± 4.37	10.8 ± 3.68	0.2	4–10
HCT, %	41.9 ± 5.3	44.3 ± 4.7	42.1 ± 4.3	0.2	F: 37–47 M: 40–54
Amylase, U/L	1443.1 ± 1005.1	911.4 ± 722.0	1526.3 ± 896.6	0.07	28–100
GGT, U/L	599.5 ± 444.3	309.2 ± 337.0	332.2 ± 406.3	0.01	F: 5–36 M: 8–61
ALP, U/L	217.9 ± 151.7	100.1 ± 65.6	142.7 ± 175.3	<0.001	F: 35–104 M: 40–129
ALT, U/L	314.5 ± 265.3	188.3 ± 475.3	169.0 ± 213.5	0.002	F: 5–33 M: 5–41
AST, U/L	257.7 ± 205.3	211.4 ± 528.2	150.1 ± 194.6	0.008	F: 5–32 M: 5–40
Glucose, mmol/L	9.0 ± 2.8	8.66 ± 3.3	7.80 ± 2.9	0.3	3.3–5.6
Urea, mmol/L	7.10 ± 3.14	5.64 ± 4.39	6.49 ± 3.17	0.049	2.76–8.07
Creatinine µmol/L	88.7 ± 31.6	94.98 ± 54.5	89.1 ± 46.3	0.5	F: 44–80 M: 62–106
Total calcium, mmol/L	2.33 ± 0.19	2.19 ± 0.27	2.31 ± 0.13	0.5	2.15–2.55

^1^ Reference ranges for laboratory tests; F indicates female reference range and M male reference range. AP: acute pancreatitis; ALP: alkaline phosphatase; ALT: alanine aminotransferase; AST: aspartate aminotransferase; BISAP: bedside index for severity in AP [21]; CRP: C-reactive protein; ERCP: endoscopic retrograde cholangiopancreatography; GGT: γ-glutamyl transferase; HCT: hematocrit; MAP: mild acute pancreatitis; MSAP: moderately-severe acute pancreatitis; SAP: severe acute pancreatitis; WBC: white blood cell count; TBA: total bile acids; B/TBA: bilirubin-to-total bile acids ratio.

**Table 2:** Statistically significant correlations between serum concentrations of TBA, B/TBA ratio, and the selected laboratory markers at 24 h from the onset of acute pancreatitis.

Variables	Spearman’s Rank Correlation Coefficients; *p* < 0.05	
	TBA	B/TBA Ratio
Bilirubin	0.56	−0.25
ALT	0.39	−0.43
AST	0.42	−0.58
ALP	0.54	−0.41
GGT	0.51	−0.37
Amylase	0.30	−0.40

**Table 3:** Diagnostic utility of TBA serum concentration and the B/TBA ratio for the diagnosis of biliary etiology of acute pancreatitis at 24 h from the onset of symptoms. For comparison, diagnostic utility of ALP, bilirubin, and GGT at the same time point is presented.

Parameter	Cut-Off Value	Diagnostic Sensitivity, %	Diagnostic Specificity, %	Diagnostic Accuracy, %	PPV, %	NPV, %
TBA, µmol/L	4.7	79	87	85	58	94
B/TBA	4.22	89	82	83	53	97
ALP, U/L	79.8	83	69	80	92	50
Bilirubin, µmol/L	40	47	85	54	92	28
GGT, U/L	282	64	69	65	89	32

PPV: positive predictive value; NPV: negative predictive value.
